# Speckle contrast of interfering fluorescence X-rays

**DOI:** 10.1107/S1600577522009997

**Published:** 2023-01-01

**Authors:** Fabian Trost, Kartik Ayyer, Dominik Oberthuer, Oleksandr Yefanov, Saša Bajt, Carl Caleman, Agnes Weimer, Artur Feld, Horst Weller, Sébastien Boutet, Jason Koglin, Nicusor Timneanu, Joachim von Zanthier, Ralf Röhlsberger, Henry N. Chapman

**Affiliations:** a Center for Free-Electron Laser Science CFEL, Deutsches Elektronen-Synchrotron DESY, Notkestrasse 85, 22607 Hamburg, Germany; b The Hamburg Center for Ultrafast Imaging, Universität Hamburg, Luruper Chaussee 149, D-22761 Hamburg, Germany; c Max Planck Institute for the Structure and Dynamics of Matter, Hamburg, Germany; d Department of Physics and Astronomy, Uppsala University, SE-75120 Uppsala, Sweden; e Institute of Physical Chemistry, Universität Hamburg, Grindelallee 117, D-20146 Hamburg, Germany; f Department of Chemistry, Fraunhofer-CAN, Grindelallee 117, D-20146 Hamburg, Germany; g Linac Coherent Light Source, SLAC National Accelerator Laboratory, 2575 Sand Hill Road, Menlo Park, CA 94025, USA; h AG Quantum Optics and Quantum Information, University of Erlangen-Nürnberg, Staudtstrasse 1, D-91058 Erlangen, Germany; i Department of Physics, Universität Hamburg, Luruper Chaussee 149, Hamburg, Germany; ESRF and Université Grenoble Alpes, France

**Keywords:** speckle contrast estimation, X-ray fluorescence, incoherent diffraction imaging, XPCS

## Abstract

With the development of X-ray free-electron lasers (XFELs), producing pulses of femtosecond durations comparable with the coherence times of X-ray fluorescence, it has become possible to observe intensity–intensity correlations due to the interference of emission from independent atoms. This has been used to compare durations of X-ray pulses and to measure the size of a focusedX-ray beam, for example. Here it is shown that it is also possible to observe the interference of fluorescence photons through the measurement of the speckle contrast of angle-resolved fluorescence patterns. Speckle contrast is often used as a measure of the degree of coherence of the incident beam or the fluctuations of the illuminated sample as determined from X-ray diffraction patterns formed by elastic scattering, rather than from fluorescence patterns as addressed here. Commonly used approaches to estimate speckle contrast were found to suffer when applied to XFEL-generated fluorescence patterns due to low photon counts and a significant variation of the excitation pulse energy from shot to shot. A new method to reliably estimate speckle contrast under such conditions, using a weighting scheme, is introduced. The method is demonstrated by comparing the speckle contrast of fluorescence observed with pulses of 3 fs to 15 fs duration.

## Abstract 2

Methods are developed to quantify the degree of coherence of fluorescently emitted X-rays from short-pulse XFEL excitations. Applications to 3 fs and 15 fs excitations are shown.

## 1. Introduction

It was recently suggested (Classen *et al.*, 2017 ▸) and demonstrated (Inoue *et al.*, 2019 ▸) that correlations of detectedX-ray fluorescence photons can be used to infer the spatial arrangements of the emitting atoms, following the principles of intensity interferometry known in astronomy (Hanbury Brown, 1968 ▸). In the classical wave picture, the correlations reveal interferences between waves emanating from the independent fluorescing sources, which can only be observed in exposures that are not considerably longer than the coherence time of those waves, equal to the fluorescence lifetime. Iron *K*α emission, for example, with a photon energy of 6.4 keV (wavelength of 1.9 Å), has a lifetime of about 0.4 fs. In the absence of detectors that can be gated with femto­second resolution, the detection of the interference of fluorescence is made possible by using short-duration pulses from an X-ray free-electron laser to excite atoms which emit waves that arrive at the detector nearly simultaneously. However, each realization of such interference will be different, due to random fluctuations of the relative phases of the emitted waves. By averaging correlations of intensities, rather than the intensities themselves, information about the unchanging structural arrangement of the fluorescing atoms can nevertheless be extracted, in a method dubbed incoherent diffractive imaging (IDI) (Classen *et al.*, 2017 ▸).

The instantaneous intensity distribution of the fluorescence is a speckle pattern formed by the sum of waves with a particular set of random phases. Even without extracting structural information, it is possible to characterize the interference of fluorescence from a measure of the speckle contrast. From this alone, one can confirm experimental conditions for IDI, compare the fluorescence lifetimes of atoms and atomic states (such as in different chemical environments or physical environments), or compare pulse durations of different operating modes of an X-ray free-eletron laser (FEL) (Inoue *et al.*, 2019 ▸). Knowledge of the speckle contrast can be used to tune the X-ray source to maximize peak brightness of pulses, or to find the location of highest intensity of a focused beam (Nakamura *et al.*, 2020 ▸).

The estimation of speckle contrast is most commonly made for diffraction patterns formed by elastic scattering rather than fluorescence. In elastic scattering the phases of atomic scattering factors are fixed (unlike for fluorescence), but the phase of the incident beam may fluctuate or the positions of the scatterers may change rapidly over the course of the exposure. In the former case, the speckle contrast provides a measure of the degree of coherence of the incident beam (Hruszkewycz *et al.*, 2012 ▸; Gutt *et al.*, 2012 ▸), and in the latter it reveals the dynamics of disordered systems (Inoue *et al.*, 2012 ▸; DeCaro *et al.*, 2013 ▸; Li *et al.*, 2014 ▸). Examples of this method, referred to as X-ray speckle visibility spectroscopy (XSVS), include the study of diffusion or vibrational modes in liquids and glasses at the atomic scale (Ruta *et al.*, 2012 ▸; Leitner *et al.*, 2009 ▸). The timescales of sample motions that are probed is dictated by the exposure time, with short-duration pulses from X-ray FELs providing access to femtosecond timescales (Hruszkewycz *et al.*, 2012 ▸). In many situations the detected signals are weak, especially when aiming for the highest time resolution and sensitivity to changes on the atomic scale. This is also certainly the case for measurements of the interference of fluorescence, which we examine in this paper. In such cases, the speckle contrast is usually obtained by averaging estimates from a number of exposures. However, as we show in this paper, that approach may lead to gross errors, especially when the incident pulse energy (number of photons) fluctuates from pulse to pulse — as for X-ray pulses created by the SASE process. Here, we introduce and examine an improved method to estimate speckle contrast, using a weighted average. We compare it with previous approaches and apply it to weak X-ray fluorescence measurements made at the LCLS from iron nanoparticles, where we demonstrate the possibility of detecting fluorescence interference at relatively low intensity.

This paper is structured as follows. The general definition of speckle contrast in Section 2 is followed by an introduction to the experiment in Section 3 with a calculation of the expected speckle contrast in Section 3.1. In Section 4 we discuss the estimation of speckle contrast and show conditions where current methods fail. Then we introduce the weighted mean speckle contrast estimation in Section 5, and apply it to the experimental fluorescence data in Section 6. We show that, using our approach, it is possible to discern an increase in the speckle contrast in data collected with a reduced X-ray FEL pulse duration, supporting efforts utilizing second-order correlations for structure determination or pulse characterization. We summarize and discuss the results in Section 7.

## 2. Speckle contrast

The origin of speckles lies in the addition of many optical waves with random phases (ϕ = [0, 2π)). This occurs when an optical laser beam is reflected from a rough surface, for example, or in the X-ray diffraction of an arrangement of atoms in a single molecule (Chapman *et al.*, 2017 ▸). For X-rays, the detected signal is proportional to the energy or square modulus of the complex-valued amplitude of the wavefield. This measurable quantity, which we refer to as the intensity, *I*, is static as long as the structure or illumination does not change. When the wavefield is spatially coherent (such as when the scattered waves are generated by a beam originating from a single point source) and strictly monochromatic, the sum of a large number of random phases most likely leads to areas of complete destructive interference where the intensity is zero. Then, the distribution of measured intensities follows a negative exponential distribution, 

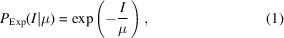

where μ is the expectation value of *I* (Goodman, 2020 ▸). Since the minimum intensity value is zero, the speckle contrast (or visibility), β, defined as the ratio of the difference between the maximum and minimum intensities to their sum, is unity [β = (*I*_max_ − *I*_min_)/(*I*_max_ + *I*_min_) = 1]. A speckle pattern can also be produced by independent emitters, emitting waves of the same wavelength but each with a random phase. In this case, the pattern will only stay constant as long as the relationships of the phases do not change, a duration referred to as the temporal coherence of the wavefield. The speckle nature of the resulting interference is, again, a consequence of the phases being random (as opposed to a phased array of emitters, for example) and follows the same distribution given in equation (1). Whether created by elastic scattering from a random substrate or from emitters with random phases, a reduction of the contrast of a speckle pattern indicates a loss of coherence of the wavefield. For example, a change in the arrangement of scatterers will change the instantaneous speckle pattern, causing intensity zeros to occur in different locations. A detector that was to integrate an exposure over the course of this change would measure the sum of these patterns. Intensity zeros in the sum would not likely occur (since this would require zeros common to both patterns) and the visibility of the measured pattern would be reduced. Likewise, a change in the relative phases of independent emitters over the course of an exposure will reduce the measured speckle contrast. The contrast of a speckle pattern formed by elastic scattering is also reduced with an incoherent source of finite extent. The effect of this reduction of spatial coherence is to convolve the speckle intensity pattern with the angular distribution function of the source (Goodman, 2020 ▸). (Finite-area pixels in a detector have the same effect.) The measurement of speckle contrast therefore provides insight into the coherence of the wavefield and from that an understanding of the nature of the emitters, the dynamics of the scatterers, or the measurement process itself. The speckle contrast can vary between β = 1, corresponding to the case of full coherence mentioned above for equation (1), and β = 0 corresponding to complete incoherence where the intensity would be uniform. However, since the light energy upon detection is quantized into countable (*x*) photons, the negative exponential distribution (β = 1) becomes a Bose–Einstein distribution (Goodman, 2020 ▸),

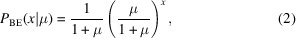

and the uniform intensity (β = 0) becomes Poisson distributed. With partial coherence, 0 < β < 1, the measured intensities follow a negative binomial distribution (Goodman, 2020 ▸),

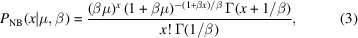

for which the variance obeys 

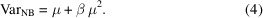




## 3. Experiment and expected speckle contrast

**Figure 1:**
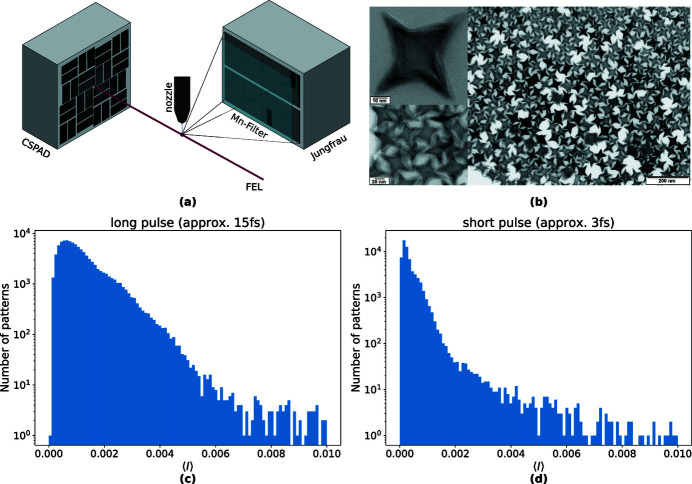
(*a*) Sketch of the experimental setup at the MFX beamline at LCLS. (*b*) Transmission electron microscope image of the iron nanoparticles. (*c*) Mean photon count per pixel at Jungfrau for 3 fs XFEL pulse patterns and (*d*) for 15 fs XFEL pulse patterns.

Measurements of iron *K*_α_ X-ray fluorescence emitted from single iron nanoparticles were carried out at the MFX beamline of LCLS, using the scheme depicted in Fig. 1 ▸(*a*). The nanoparticles, referred to as iron nano-stars (Feld *et al.*, 2019 ▸), had an irregular but roughly spherical shape with a mean diameter of about 50–100 nm, see Fig. 1 ▸(*b*). These samples were suspended in toluene at a concentration of 0.13 mol l^−1^ (7.9 × 10^19^ ml^−1^) and injected across the focused X-ray beam as a liquid jet. The jet, formed by a double-flow-focusing nozzle (Oberthuer *et al.*, 2017 ▸; Knoška *et al.*, 2020 ▸), had a diameter of about 2.2 µm and a velocity of about 60 m s^−1^, ensuring that a fresh sample was present for each exposure, made at a repetition rate of 120 Hz. The LCLS was operated in two different modes for the measurements, to produce pulses of ∼15 fs, as estimated using the X-band Transverse Deflecting Cavity (XT-CAV) (Krejcik *et al.*, 2013 ▸), and ∼3 fs, as estimated by settings of the electron pulse compression in the accelerator. The incident X-ray beam was linearly polarized in the horizontal direction with a photon energy of 7.15 keV and focused to a size of about 4 µm. From estimates of the beamline transmission, the mean pulse energy at the experiment was about 0.1 mJ for the short and 1.5 mJ for the long pulse mode. Thus the peak X-ray intensity on the sample approached 8 × 10^17^ W cm^−2^ for the long pulses and 2.7 × 10^17^ W cm^−2^ for the short ones. The fluorescence was measured using a Jungfrau detector oriented at a scattering angle of 90° in the horizontal plane, where coherent scattering is minimized. The detector, with 1000 × 1000 square pixels, each 75 µm wide, was placed 120 mm from the interaction region. A 32.4 µm-thick manganese filter was placed in front of the detector to attenuate the iron *K*_β_ fluorescence and any coherently scattered photons.

The concentration of the nanoparticles in the solution was adjusted so that on average 11% of the pulses intersected a particle. This ‘hit fraction’ was measured simply from the sum of fluorescence counts on the Jungfrau detector, monitored on-line using the program *OnDA* (Mariani *et al.*, 2016 ▸). After the experiment, the frames containing fluorescence counts were processed by first masking bad pixels and shadows of shielding around the edges, leaving 895000 pixels per frame. The treatment of each detector frame to yield photon counts is described in Appendix *F* [App appf]. The number of events (frames with detected fluorescence) was 98000 and 61000 for the ‘long’ 15 fs and ‘short’ 3 fs exposure times, respectively. Histograms of the mean number of photons per pixel, 〈*I*〉, in each event for the long and short pulses are given in Figs. 1 ▸(*c*) and 1 ▸(*d*), respectively. It is seen that in both cases the mean counts are less than one photon per 100 pixels, but this varies considerably over both datasets. The large variation of mean counts was in part due to the sample delivery — the nanoparticles arrive randomly in the beam focus — and in part due to the fluctuation of the pulse energy of the XFEL beam.

### 3.1. Expected speckle contrast

Under the assumption of a Gaussian-shaped excitation pulse, where the pulse duration (FWHM) *T* is significantly greater than the coherence time τ_c_, the expected speckle contrast is well approximated by 

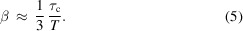

A derivation of equation (5) can be found in Appendix *A* [App appa]. The coherence time can be estimated from the spectral line-width. For iron *K*_α_, with a line-width (FWHM) of Γ = 1.61 eV (Krause & Oliver, 1979 ▸), τ_c_ = 2ℏ/Γ = 0.8 fs (Grynberg *et al.*, 2010 ▸; Goodman, 1985 ▸; White, 1934 ▸). In these measurements, *K*_α,1_ and *K*_α,2_ fluorescence cannot be discriminated, and these will contribute as mutually incoherent modes, with the ratio given by the fluorescence branching ratio 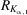 = 0.581 and 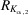 = 0.297 (Brunetti *et al.*, 2004 ▸), and thus 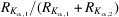 ≃ 2/3 and 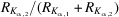 ≃ 1/3. The probability that two detected photons can interfere is therefore reduced by a factor (2/3)^2^ + (1/3)^2^ = 5/9 (Lohse *et al.*, 2021 ▸). Based on these considerations, the maximum speckle contrast that can be expected is 

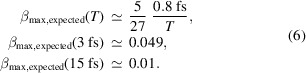

There are, however, a number of factors which act to reduce the achievable speckle contrast below the estimates of equation (6), discussed qualitatively here:

*Insufficient speckle sampling*. The speckle size of the pattern measured at the detector is inversely proportional to the illuminated sample size. If this is smaller than the detector pixel size, contrast is reduced (Goodman, 1975 ▸). Given the 75 µm pixel size located 120 mm from the sample, the maximum sample diameter for sufficient speckle sampling is 300 nm. For particle diameters of 300 nm the contrast is reduced by a factor of 0.74. The iron nanoparticles used in this experiment had a diameter of 50–100 nm, for an expected reduction in β by a factor of 0.95 to 0.93.

*Background*. Despite the use of the Mn filter and the choice of a 90° scattering angle, we estimate a background of less than 5% of the total signal that is not attributable to fluorescence of the sample. Some of this may be caused by Mn fluorescence in the filter. Such a background reduces the contrast by a factor of >0.95.

*Finite speed of light*. The arrival times of fluorescence at the detector varies even for an instantaneous X-ray pulse, due to the spatial extent of the sample. This effect is most severe for our 90° scattering geometry and is reduced for detection in the forward direction (Lohse *et al.*, 2021 ▸; Shevchuk *et al.*, 2021 ▸). Simultaneously generated fluorescence will only interfere if the path difference to the detector is less than the product of the speed of light with the coherence time, equal to 120 nm — very close to the size of the particles used.

*Ionization and plasma effects*. The intense X-rays pulses are expected to lead to high ionization and formation of plasma in the sample, which could affect the fluorescence. For the conditions in the experiment, we simulated the ionization dynamics in the sample using a non-thermal plasma approach (Jönsson *et al.*, 2017 ▸) and found that the short pulse (3 fs, 0.1 mJ) leads to sample temperatures of 1.5 eV and an average ionization for iron of 0.1, while the long pulse (15 fs, 1.5 mJ) gives a temperature of 3.5 eV and an average ionization for iron of 2. The simulated spectra do not show broadening for these conditions; however, the plasma effects could become significant at higher intensities.

## 4. Estimation of speckle contrast

The low mean photon counts of our measurements is a situation not uncommon in the analysis of X-ray speckle patterns, most of which are made under the conditions of very limited signal levels. This is certainly the case in the field of X-ray photon correlation spectroscopy (Lehmkühler *et al.*, 2021 ▸) since the study of dynamics of samples requires short exposures, and the brightness of any X-ray source is ultimately limited. Under these conditions of low signal levels where only single photons are detected instead of visible speckles, the definition of speckle contrast given in Section 2 is not practical. Given that the measured counts follow the negative binomial distribution of equation (3), the most straightforward method to determine the speckle contrast of a low-signal pattern is to estimate μ from 〈*I*〉 and the variance Var(*I*) from the square of the standard deviation of the intensity values, *I*. Then, simply solving equation (4) for the visibility factor yields 

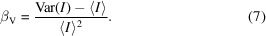

We call the speckle contrast estimated this way β_V_.

Another approach is to count the detector pixels that measured one or two photons (Hruszkewycz *et al.*, 2012 ▸; Möller *et al.*, 2019 ▸; Sun *et al.*, 2020 ▸). Given the measured observed frequency of one-photon values, *P*_1_ = *P*_NB_(1|〈*I*〉, β), and two-photon values, *P*_2_, we find from equation (3)


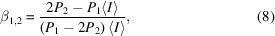

where the subscripts 1 and 2 stand for the use of only 1 and 2 photon counts. Note that for μ = 1, β_1,2_ is not defined, since *P*_1_ = 2*P*_2_ | ∀ β. This estimate does not appear to have any advantage over β_V_ but has some significant disadvantages when the mean photon count approaches or exceeds 1, as we will show below. Since approximated forms of β_1,2_ are often mentioned in the literature (Hruszkewycz *et al.*, 2012 ▸; Sun *et al.*, 2020 ▸; Möller *et al.*, 2019 ▸) (see Appendix *B* [App appb]), we include it in our further analysis. Furthermore, speckle contrast estimation requires a minimum number of simultaneously measured values (*e.g.* pixels). Even though this was not a concern in our experiments, the effects of an insufficient pixel count are discussed in Appendix *C* [App appc].

Applying equations (7) or (8) to each of the 61000 short-pulse patterns and separately to each of the 98000 long-pulse patterns, then averaging the results, we obtain the following speckle contrast estimates,

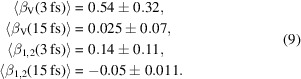

**Figure 2:**
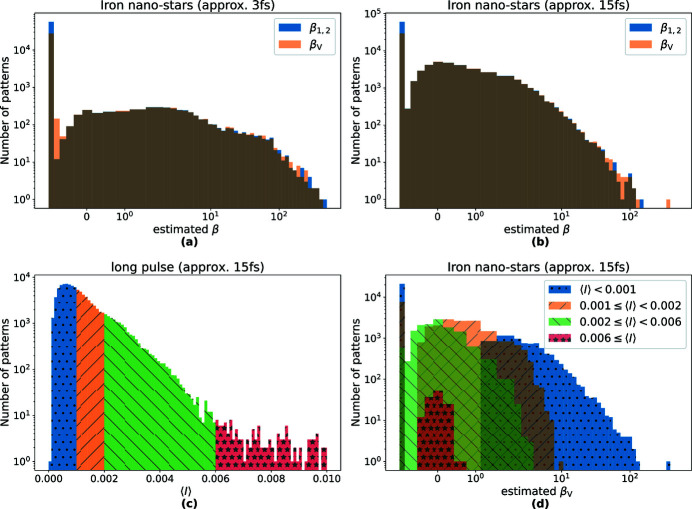
Histograms of single pattern speckle contrast estimates β_1,2_ and β_V_, obtained from measured X-ray fluorescence photons. (*a*) Iron nano-stars, measured with 3 fs pulses, with the sample variance Var(β_1,2_) = 708 and Var(β_V_) = 6253. (*b*) Iron nano-stars, measured with 15 fs pulses, with Var(β_1,2_) = 11 and Var(β_V_) = 485. Note the long-tailed distribution with many entries at −1 and some quite high β estimates, along with the high sample variance. (*c*) Histogram of mean photon counts [equivalent to Fig. 1 ▸(*d*)], divided into four parts as indicated by four colors. (*d*) Histograms of speckle contrast estimates for different regions of mean photon counts. Note the transition from a long-tailed distribution with large peak at β = −1 at low 〈*I*〉 (blue) to a more Gaussian-like distribution for higher 〈*I*〉 (red).

These estimates are much higher than the optimistic expectations of equation (6), except for the negative value for β_1,2_ at 15 fs. The largest estimate is unphysical since a speckle contrast of β = 0.5 corresponds to perfectly coherent but unpolarized light. Also, the differences between 〈β_V_〉 and 〈β_1,2_〉 are quite large. These estimates therefore cannot be trusted, and the reason for this is the very low mean photon count for the vast majority of patterns and also especially the large variation of 〈*I*〉 from pattern to pattern as evident in Figs. 1 ▸(*c*) and 1 ▸(*d*). Histograms of the individual estimates are shown in Figs. 2 ▸(*a*) and 2(*b*) for the short and long pulses, respectively. The abscissa of both plots is logarithmic, highlighting the long-tailed distribution of these single pattern speckle contrast estimations, and which severely skews the means given in equation (9).

Hints for how to find better estimates of the contrast can be found by examining subsets of patterns chosen from various bins of 〈*I*〉 in the histogram of Fig. 2 ▸(*c*). We find that the distributions of β estimates — and especially the behavior of the long tail at high values — depends on the mean counts 〈*I*〉, as seen in Fig. 2 ▸(*d*). While a low 〈*I*〉 leads to a large fluctuation of β estimates, this transmutes to a more compact Gaussian-like distribution for larger 〈*I*〉 values. This observation led us to propose a new method to estimate speckle contrast, using a weighted average as described and evaluated, using simulated data, in the next section.

## 5. Weighted mean speckle contrast

To evaluate our new strategy to estimate the speckle contrast of patterns with low photon counts per pixel, 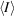 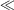 1, and with a large variation in 〈*I*〉 from pattern to pattern, we first simulated 10^5^ speckle patterns with a mean count of 10^−4^, 10^5^ more patterns with a mean count of 3 × 10^−4^, and 1.5 × 10^4^ speckle patterns with a mean count of 3 × 10^−2^. This was done simply by generating random numbers that follow the Bose–Einstein distribution of equation (2), corresponding to full contrast (β_0_ = 1). Each pattern consisted of one million pixels — that is, one million random numbers — similar to that of our experiment and sufficiently large to ensure that the mean estimate converged to the correct value, as demonstrated in Appendix *C* [App appc].

**Figure 3:**
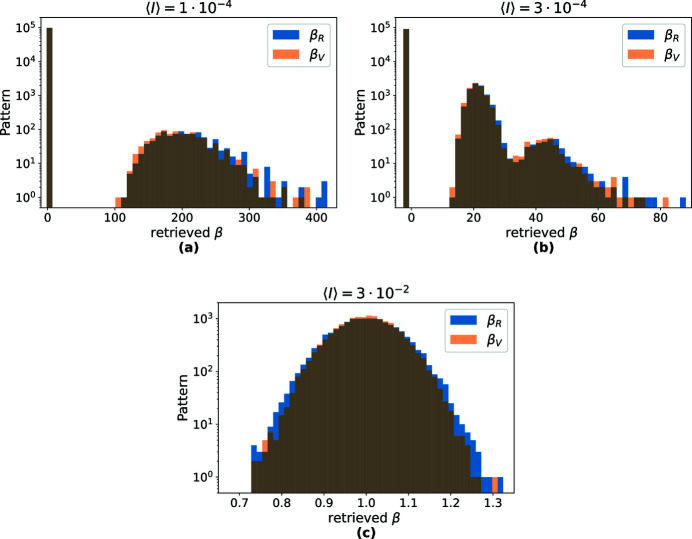
Histograms of speckle contrast estimates β_1,2_ and β_V_, obtained from simulated patterns each consisting of one million random numbers following a Bose–Einstein distribution (β_0_ = 1) and with means of (*a*) 〈*I*〉 = 10^−4^, (*b*) 〈*I*〉 = 3 × 10^−4^ and (*c*) 〈*I*〉 = 3 × 10^−2^. For (*a*) and (*b*) most of the estimates are at β = −1 and a minority at very high values.

Histograms of the per-pattern speckle contrast estimates are plotted in Fig. 3 ▸ for the three different mean photon counts. For the lowest signal level of 10^−4^ counts per pixel (corresponding to an average of only 100 photons per pattern) the probability of observing at least one two-photon hit within a single pattern is very small. Thus, most simulated patterns do not have any pixels with a value of 2 or higher. In this case, *P*_*j* ≥ 2_ = 0 and *P*_1_ = 〈*I*〉, and therefore Var(*I*) = 〈*I*〉 − 〈*I*〉^2^, so that equation (7) evaluates to β_V_ = −1. Likewise, equation (8) with *P*_2_ = 0 immediately returns β_1,2_ = −1. These values occur frequently for 〈*I*〉 = 10^−4^ and 3 × 10^−4^ as seen in the histograms of Fig. 3 ▸. Conversely, a pattern containing at least one pixel with a value of 2 or higher will return an overly large β estimate, using equations (7) or (8).

From Fig. 3 ▸ it is apparent that the shape of the distribution of β estimates changes with 〈*I*〉. In Fig. 3 ▸(*a*), with 〈*I*〉 = 10^−4^, most entries are at β = −1 and a few entries are distributed over a wide range of large β values. At a slightly higher 〈*I*〉 = 3 × 10^−4^, shown in Fig. 3 ▸(*b*), this transmutes to a distribution consisting of peaks (caused by patterns with one two-photon value, two two-photon values, and so on). Finally the distribution takes on a Gaussian shape, centered at β_0_ for sufficiently large 〈*I*〉, as seen in Fig. 3 ▸(*c*). Despite the differences in the distributions, the averages of the β estimates in each of the cases presented in Fig. 3 ▸ all have the correct value of 1 (equal to β_0_). However, this is only true when averaging over patterns with the same 〈*I*〉. With significant intensity fluctuations, β estimates are averaged over values sampled from significantly different distributions. It is unlikely in that case that the β ≫ 1 estimates that are obtained in patterns with two-photon counts will be properly balanced by the β = −1 estimates obtained when there are no two-photon counts. This observation suggests that it may be unwise to apply equal weightings to estimate β from patterns with different 〈*I*〉. To obtain reliable speckle contrast values from data sets with varying mean intensities, we therefore suggest forming the weighted mean of the single pattern β-estimates using the inverse of their expected variances as weights,

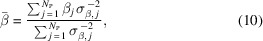

with *N*_P_ denoting the number of patterns, β_*j*_ the estimated speckle contrast of the *j*th pattern and 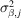 the expected variance of β_*j*_(〈*I*〉, β_0_). The variance of the weighted mean speckle contrast is then given by 

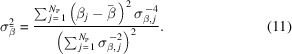

However, to apply this weighting, we need to know the expected variance of each β_*j*_, namely 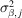 . In the following, we derive and examine schemes for evaluating weighted averages of β_1,2_ and β_V_.

### 5.1. Weighted mean of β_1,2_

As derived in Appendix *D* [App appd], the variance 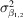 of β_1,2_ can be expressed as 

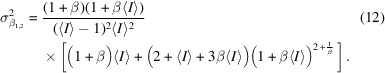

**Figure 4:**
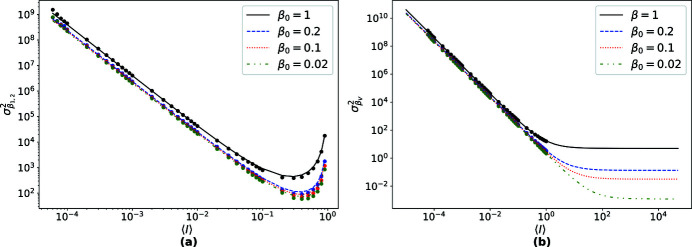
(*a*) Variance of β_1,2_ as a function of 〈*I*〉 as computed using equation (12) (solid lines) and simulated values (dots). The variance decreases with increasing 〈*I*〉 for low signals and then increases again as 〈*I*〉 approaches 1. (*b*) Variance of β_V_ as a function of 〈*I*〉 as computed using equation (13) (solid lines) and simulated values (dots). Note that 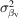 saturates at high 〈*I*〉.

This is plotted as a function of 〈*I*〉 as solid lines in Fig. 4 ▸(*a*) for several true values of the speckle contrast, β_0_. To verify this expression, calculations were also carried out on simulated data. As for the simulations above, sets of 10^6^ random numbers were generated following a negative binomial distribution, corresponding to patterns recorded with a 1 megapixel detector. Groups of patterns were simulated for constant β_0_ and 〈*I*〉, for values of 〈*I*〉 spanning 5 × 10^−3^ to 1. The number of simulated patterns per group decreased from 10^5^ for the smallest 〈*I*〉 to 5000 patterns for the largest. For each pattern, β_1,2_ was calculated using equation (8) from which sample variances were determined and plotted as dots in Fig. 4 ▸(*a*). As seen in that figure, the theoretical and simulated variances 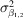 are in good agreement. Small deviations between them can be explained by the fact that the assumed independence of the observables *P*_1_, *P*_2_ and 〈*I*〉 is slightly violated given that there is a finite number of pixels.

**Figure 5:**
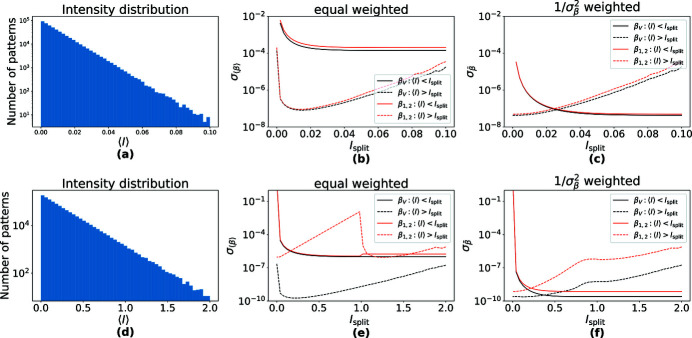
Effects of 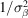 -weighting demonstrated on simulated data in which the mean counts per pattern follows exponential distributions with (*a*) mean 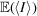 = 0.01 and (*d*) 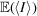 = 0.2. The data were divided into two parts: one at high intensity with 〈*I*〉 > *I*_split_, and its complement with 〈*I*〉 ≤ *I*_split_. (*b*, *e*) Standard deviation of the retrieved β of the two parts, using equal weighting, as a function of *I*_split_. The standard deviation decreases when neglecting the patterns with very low counts as evident in the low-intensity regime of (*b*). The plot of 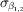 in (*e*) exhibits a sharp discontinuity at *I*_split_ = 1, which is absent for 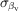 . (*c*, *f*) Standard deviation of the retrieved β of the two parts, using 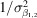 -weighting. In this case the lowest standard deviation is achieved by using all patterns to estimate β. β_V_ always performs better than β_1, 2_, especially in the high-intensity regime, see (*f*).

We next simulated 5 × 10^5^ patterns of 1 megapixel size and with β_0_ = 1, but now with fluctuating mean counts. The mean counts 〈*I*〉 for each pattern were chosen randomly from a negative exponential distribution with the expectation-value 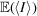 = 0.01. A histogram of these is given in Fig. 5 ▸(*a*). This distribution corresponds to a SASE process with a single mode, for example, yielding measurements with an average of 0.01 counts per pixel per pattern and a maximum value of 〈*I*〉 = 0.1. For each simulated pattern, β_1,2_ was calculated using equation (8). To examine the effectiveness of the inverse variance weighting, the patterns were divided into two subsets depending on whether 〈*I*〉 was smaller or larger than a particular threshold, *I*_split_.

For both the low-intensity and high-intensity subsets obtained for various choices of *I*_split_, we calculated the weighted mean 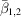 and its standard deviation 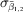 , as well as the unweighted mean 〈β_1,2_〉 and its standard deviation. The standard deviations 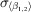 of the unweighted means for the low-intensity and high-intensity subsets are plotted as a function of *I*_split_ as the red solid line and red dashed line, respectively, in Fig. 5 ▸(*b*). The inverse variance-weighted standard deviations 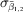 for the two subsets are plotted in Fig. 5 ▸(*c*), also as red solid and red dashed lines.

Comparing the red lines in Fig. 5 ▸(*b*) with those in Fig. 5(*c*) shows reductions of the standard deviations for both the low-intensity and high-intensity subsets when applying the weighting scheme. This improvement is also apparent when using the entire set of patterns, as when the threshold of the low-intensity subset is equal to the maximum value of *I*_split_ = 0.1 or (equivalently) for the high-intensity subset at *I*_split_ = 0. In this case the weighting scheme yields a standard deviation of 4 × 10^−8^, compared with 10^−4^ for the unweighted mean. It is also noted that the unweighted 〈β_1,2_〉 of the high-intensity bin (〈*I*〉 ≥ *I*_split_, red dashed line) becomes worse if intensity data with a mean lower than about 0.01 are included. That is, the unweighted mean 〈β_1,2_〉 suffers from a higher uncertainty when all data are included compared with when the very low intensity patterns are neglected. With inverse variance-weighting, on the other hand, including all data, no matter how low the mean counts, the uncertainty of the mean 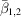 reduces.

It may seem circular that we need β to calculate 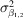 , which is then used to determine 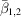 , but it turns out that exact knowledge of β is not crucial and an initial guess can be used to recursively determine 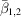 .

To put things into perspective, in the given example the standard deviation of the mean unweighted β is about 3500 times higher than that of the weighted mean, considering the full dataset. This means that, in order to obtain a similar accuracy, 1.2 × 10^7^ times as many patterns would be required. However, when discarding the low-photon-count data (in the present case around 76% of the whole dataset), the standard deviation can be reduced by a factor 3 × 10^−4^. Now the difference to the weighted case is quite small, but the accuracy stays lower.

### 5.2. Weighted mean of β_V_

An evaluation of the inverse variance-weighting of 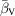 was performed similarly to the case of 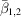 presented in Section 5.1. The variance of 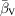 , required for the weighting, is given as 

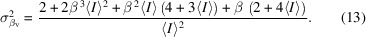

A detailed derivation of this equation can be found in Appendix *E* [App appe], and a verification of the expression is presented in Fig. 4 ▸(*b*) utilizing the same simulated datasets as in Section 5.1.

Plots of the variances of estimates of β_V_ are given in Figs. 5 ▸(*b*) and 5(*c*) for the low-intensity and high-intensity dataset fractions, as the black solid and black dashed lines. The variances are quite similar to those observed for β_1,2_.

Differences in the β_V_ and β_1,2_ methods only become apparent for mean counts higher than about 0.1. To investigate these, we simulated a set of patterns with β_0_ = 1 and an exponential distribution of mean photon counts but with a higher expectation value of 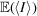 = 0.2 and a maximum of 2.0 photons per pixel per pattern. A histogram of the mean counts per pattern is plotted in Fig. 5 ▸(*d*). The variance 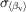 of the equal-weighted 〈β_V_〉 is plotted in Fig. 5 ▸(*e*) (black solid and dashed lines) as a function of *I*_split_ for the low-intensity and high-intensity dataset subdivisions. Calculations were also made on this dataset using the β_1,2_ method. The plots of the variances of 〈β_1,2_〉 (red solid and dashed lines) show a critical behavior around *I*_split_ = 1, which is due to the definition gap of β_1,2_ at 〈*I*〉 = 1.

The accuracy of the equal-weighted 〈β_V_〉 decreases when we take the low 〈*I*〉 into account [as apparent from the black dashed line in Fig. 5 ▸(*e*)], similar to the case of the equal-weighted 〈β_1, 2_〉, as discussed before. The standard deviations of the inverse variance-weighted 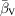 (black lines) and 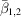 (red lines) are plotted in Fig. 5 ▸(*f*), both showing a significant improvement as compared with the unweighted averages. While for low photon count data the accuracy of 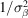 -weighted 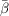 is almost the same for β_1,2_ and β_V_, the latter is significantly better for high photon counts. We can state, as an intermediate conclusion, that the 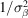 -weighted 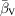 approach is preferable when retrieving the speckle contrast from data consisting of patterns with different mean photon counts.

## 6. Speckle contrast determination of *K*_α_ – X-ray fluorescence

We can now apply our proposed 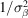 weighting of speckle contrast estimates on the experimental fluorescence data described in Section 3. Utilizing equation (10) we obtain 

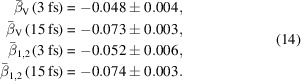

In contrast to the unweighted values in equation (9), the values of 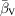 and 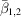 are in much better agreement. The estimated speckle contrast is negative, which would imply a sub-Poissonian photon distribution which is not expected. The result can be explained by systematic errors of the photon­ization method used to extract photon counts from the measured detector frames (see Appendix *E* [App appe]), and in particular in the discrimination of one-photon and two-photon hits. In our case, the photonization algorithm underestimates the two-photon hits in favor of the one-photon hits, which leads to a systematic underestimation of the retrieved speckle contrast. Due to this bias it is therefore not possible to obtain the speckle contrast absolutely. Sun *et al.* (2020 ▸) recently discussed such systematic errors in the estimates of speckle contrast induced by the photonization algorithms. They demonstrated that the error behaves linearly for measurements with small mean photon counts 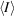 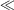 1, implying that the difference of the retrieved speckle contrast for different coherence conditions could be trusted. We therefore report the retrieved speckle contrast difference 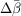 = 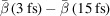 instead of the absolute values, as meaningful results,

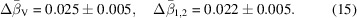

The maximum expected speckle contrast difference due to the change in incident X-ray pulse duration using equation (6) is Δβ_0_ = 0.039. However, as detailed in Section 3.1, there are many experimental factors that will reduce the contrast, and so too will reduce the contrast difference. Our estimated 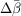 is therefore consistent with the changes in pulse duration and is consistent with the interference of fluorescence photons as the cause of the speckle contrast.

## 7. Summary

The speckle contrast, or visibility, of an intensity pattern of electromagnetic radiation quantifies the degree of coherence or number of coherent modes present, and can be determined from intensity measurements using a pixel array detector, even when the detected photon counts are much less than one per pixel. We aimed to measure the speckle contrast of *K*_α_ iron X-ray fluorescence emitted from single iron nanoparticles by short-duration pulses from an X-ray free-electron laser (XFEL). When the pulses are not significantly longer than the coherence time of the fluorescence (as set by the lifetime of the fluorescence), it is expected that the emission from different atoms will interfere at the detector, providing a possible route to image the atomic arrangements. We examined the speckle contrast of the intensity measurements to investigate the potential of discerning such interferences and hence for carrying out such imaging of small samples.

In our experiment, the fluorescence was recorded using X-ray free-electron laser pulses of 3 fs duration and of 15 fs duration. 61000 patterns were recorded for the short-pulse configuration and 98000 for the long-pulse configuration. The patterns, recorded on a pixel-array detector with almost a million pixels, were very sparse, containing on average 9 × 10^−4^ photons per pixel per pattern. The speckle contrast could only be measured by averaging over a large number of patterns. However, in doing so, we discovered that existing methods to estimate the speckle contrast fail for sparse patterns when there are large variations in the strength of the patterns from pulse to pulse, as was the case here. The reason for this failure was tracked down to the fact that the distribution of estimates of the speckle contrast, for a given mean photon count per pixel 〈*I*〉, changes dramatically for different 〈*I*〉. To overcome this problem we proposed a new way to estimate speckle contrast, by calculating the average of estimates weighted by the inverse of the expected variance of those estimates. Using simulated data, we showed that this approach produces the correct results when previous approaches did not, see equation (15).

Using this approach of inverse variance weighting, we observed a larger speckle contrast for patterns recorded with the 3 fs short pulses than for the 15 fs pulses. This result indicates that the speckle contrast of X-ray fluorescence emitted from small particles can be measured when illuminated by short pulses, and these measurements could be used to characterize XFEL pulses shorter than about 10 fs. However, that would require careful calibration, for example by measuring a sample with no expected speckle contrast (β = 0) as a reference, and a well controlled sample size for constant speckle sampling. Our results support the finding of Inoue *et al.* (2019 ▸) that X-ray fluorescence can be used for imaging based on intensity correlations, as proposed by Classen *et al.* (2017 ▸).

The presented method of weighted speckle contrast estimation might also be useful for XSVS experiments with a low and non-constant mean photon count per exposure. This might especially be the case at XFELs due to the fluctuating intensity between X-ray pulses, or due to a serial deployment of the specimens.

## Figures and Tables

**Figure 6:**
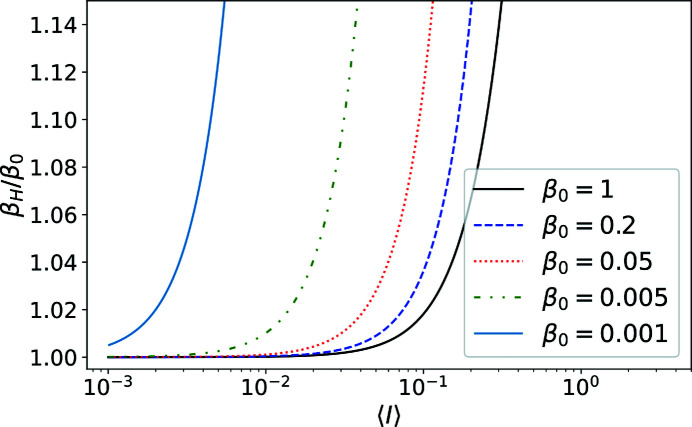
Plot of the estimated speckle contrast β_H_ as a function of the mean counts. β_0_ represents the true visibility. The approximation is worse when the true speckle contrast β_0_ is weaker.

**Figure 7:**
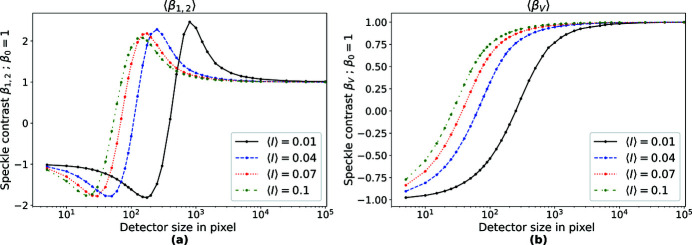
Influence of the detector size on the estimated speckle contrast using (*a*) β_1,2_ and (*b*) β_V_. For each 〈*I*〉 a set of 5 × 10^8^ random numbers was generated that follow the distribution of equation (2). These were distributed into sets according to the number of pixels in the detector.

**Figure 8:**
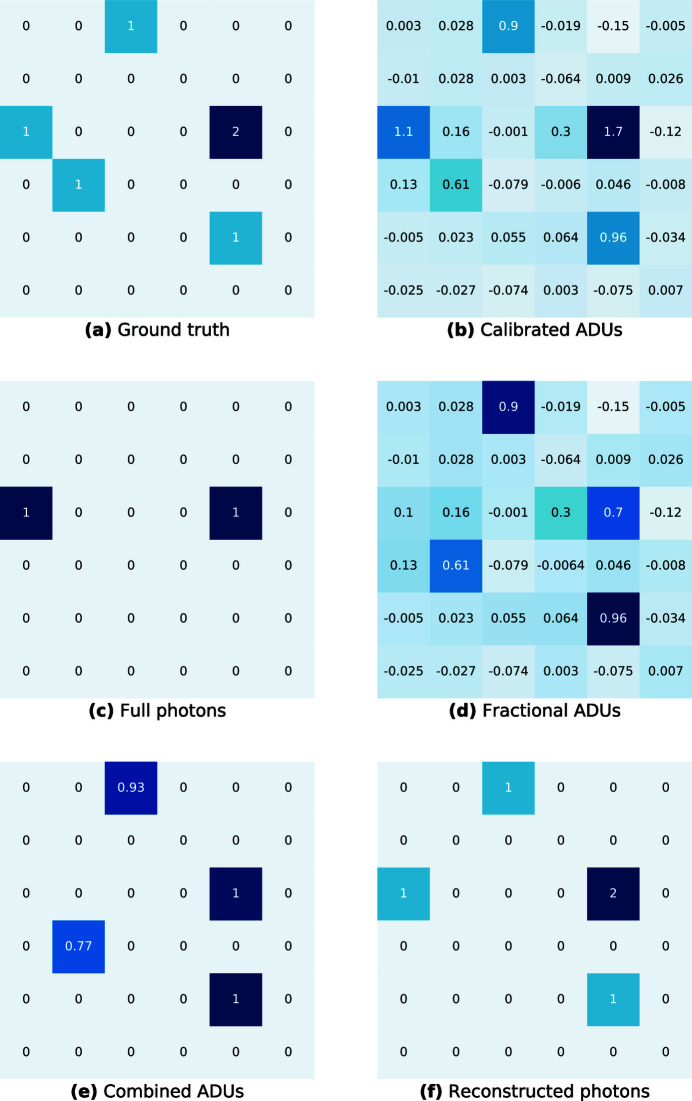
Illustration of the LAP photonization algorithm.

## Appendix A Fluorescence speckle contrast for a Gaussian excitation pulse

For the following discussion we assume that the radiative lifetime is not longer than the coherence time of the emission. We further neglect the unpolarized nature of fluorescence for now, and assume that there is only one decay path (single spectral line). This means that in the case of an infinitely short excitation pulse we expect a speckle contrast of 1. In that case the wavefunction of the emission of frequency ω_0_, excited at *t* = 0, can be expressed as (White, 1934 ▸)

and the intensity is given by    accordingly, where the step function Θ(*t*) = 1 for *t* ≥ 1 and 0 otherwise. The modulus of the complex degree of coherence represents the probability of the ability of two waves to interfere, given by (Goodman, 1985 ▸)

The coherence time is calculated from the complex degree of coherence by (Goodman, 1985 ▸)

thus identifying the parameter τ_c_ with this quantity. The excitation probability is proportional to the incident XFEL pulse intensity *I*_excite_(*t*), and we define the normalized pulse shape  =  . The expected speckle contrast is obtained as (Inoue *et al.*, 2018 ▸; Goodman, 1985 ▸)

where  =  is the autocorrelation of the normalized excitation pulse shape. When we assume a Gaussian-shaped excitation pulse,

with a FWHM *T*, then equation (19) evaluates to

where erf(*x*) denotes the error function. The visibility becomes unity in the limit of the excitation pulse being much shorter than the coherence time: β_Gauss_ → 1 as τ_c_/*T* → ∞. In the limit of the pulse duration being significantly longer than the coherence time, which is the case in our experiment, the speckle contrast becomes

In the case of unpolarized light, as is the case for fluorescence detected without a polariser, the speckle contrast is halved (Goodman, 1985 ▸), such that

## Appendix B Further approximation of β_1,2_

The most common approximation of β_1,2_ is to neglect values of three or more photons when estimating the mean photon count (*P*_≥3_ = 0 ⇒ 〈*I*〉 = *P*_1_ + 2*P*_2_) (Hruszkewycz *et al.*, 2012 ▸; Sun *et al.*, 2020 ▸; Möller *et al.*, 2019 ▸). Furthermore, a Taylor series expansion around *P*_2_ = 0 yields

For example, Hruszkewycz *et al.* (2012 ▸) truncated equation (24) to define an estimate,

It is not apparent what the advantage of this approach is. Plots of β_H_ calculated from equations (25) and (3), normalized by the true visibility β_0_, are shown in Fig. 6 ▸. Although this estimate does not diverge at 〈*I*〉 = 1, as does β_1,2_, it nevertheless becomes more inaccurate as 〈*I*〉 increases to 1, for all values of β_0_. The plot also reveals an undesired dependence of β_H_ on the true visibility β_0_.

## Appendix C Influence of the detector size on speckle contrast estimation

Here we discuss the influence of the number of detector pixels on the accuracy of speckle contrast estimation. The estimate β_1,2_ requires that the probabilities for detecting one and two photons (*P*_1_ and *P*_2_), as well as the value of 〈*I*〉, can be estimated sufficiently independently. To illustrate the need for many pixels, consider the extreme case of a two-pixel detector. Obviously, it is impossible to obtain β_1, 2_ > 0 because this would require either  or  , which cannot happen.

To further analyze the influence of the pixel count, we performed numerical simulations by generating sets of random numbers that follow the Bose–Einstein distribution of equation (2) (that is, β = 1) with different means μ for each set. Each set consisted of 5 × 10^8^ generated numbers. The values in each set were then distributed into groups of equal sizes, corresponding to the number of detector pixels. The speckle contrast was evaluated for each of these ‘detector frames’ individually and averaged afterwards to obtain an estimate of β_1,2_ or β_V_ for a particular mean intensity 〈*I*〉 (given by the mean counts in the set). These estimates, plotted in Fig. 7 ▸ as a function of detector size, were therefore all obtained using the same number of total pixel readings, since a smaller detector size gives more detector frames to average.

It is seen from Fig. 7 ▸(*a*) that in the limit of detectors with few pixels the contrast estimate approaches β_1,2_ = −1. The correct value is only obtained for pixel counts above about 10^4^. We note two properties: for lower 〈*I*〉, more pixels are required in order to obtain an accurate estimate, and an insufficient pixel count can lead to an overestimation of the contrast (β_1,2_ > β_0_).

The same evaluation for β_V_ yields the results illustrated in Fig. 7 ▸(*b*). Also here, the limit of detectors with few pixels leads to an underestimation of β_V_, but in contrast to β_1,2_ it converges monotonically to the true value as the pixel count increases.

## Appendix D Derivation of Var(β_1,2_)

In order to calculate  we make use of the linear error propagation approximation, as given by

To obtain  we need  ,  and  . Therefore, we assume a sufficiently large detector, where sufficiently large means that *P*_1_ and *P*_2_ can be obtained acceptably independent from each other. A trivial negative example would be a one-pixel detector, where we have no chance of obtaining a one-photon and a two-photon hit within a single measurement. That might sound very obvious but we would like to emphasize that our treatment requires that all *P*_*j*_ are independently measurable observables. Thus *P*_*j*_ is found simply by counting *j*-photon events and then dividing the count by the number of pixels *P*_*j*_ = *n*_*j*_/*N*_Pix_. This is a counting process that satisfies the Poisson process and therefore we obtain

We have ignored the factor 1/*N*_Pix_ since it is constant and applies equally to all terms. The last required quantity, the variance  , is given by  =  .

Now equation (26) can be applied to equation (8), and we obtain

## Appendix E Derivation of Var(β_V_)

Here we derive  in an analogous way to  . Therefore we express the mean number of counts as a function of the photon probability, which reads

and its variance

With this we can write β_V_ as a function of {*P*_*j*_} as

As in Appendix *D* [App appd], we assume that all *P*_*j*_ follow Poisson statistics, since they originate from countable observables, and thus the variance of β_V_, in the linear error propagation approximation, is given by

To evaluate this equation, we need to differentiate equation (31) with respect to *P*_*j*_ | ∀_*j*_,

Now we can express equation (32) as a function of 〈*I*〉 and β,

## Appendix F Photonization of detector values

The data, measured with a Jungfrau detector, were calibrated as follows. We started with the pre-calibrated data provided by *psana* (Damiani *et al.*, 2016 ▸). Instead of using dark runs, the noise peak of the histogram of data values was fitted for each pixel and each run separately and used to generate maps of the dark noise (so-called dark fields), for each run individually. This was possible since all recorded images were sparse (with photon counts  1 per pixel). This dark calibration procedure is slightly better than measuring dark runs since the dark current drifts over time and thus may differ at the times of the measurements. Since we were only interested in the iron *K*_α_-fluorescence at 6.4 keV photon energy, the detection of photons of different energies was minimized by using a metal-foil filter and a scattering angle where elastic scattering is a minimum. We fitted the 6.4 keV peak for each pixel (using all available data) and calibrated each pixel (assuming a sufficiently linear behavior of the Jungfrau detector) such that an ADU value for one 6.4 keV photon was re-scaled to 1.0.

To take care of charge sharing and to assign an integer photon count for each pixel we made use of a method we call ‘largest adjacent pixel (LAP)’ and which is equivalent to the Psana Photon Converter described by Sun *et al.* (2020 ▸). This method is presented as an example in Fig. 8 ▸ on some arbitrary values, where Fig. 8 ▸(*a*) represents the ground truth and Fig. 8 ▸(*b*) the detector values (ADUs). In the first step, the value is split into whole (integer) numbers, see Fig. 8 ▸(*c*), and the remaining fractional values [Fig. 8 ▸(*d*)]. Using the assumption that charge sharing only occurs between two adjacent pixels (sharing an edge), we select pixels above a certain value (here *x*_seed_ = 0.5) and combine each with their largest adjacent pixel [Fig. 8 ▸(*e*)]. If the combined value exceeds a certain threshold (here *x*_trh_ = 0.8), the pixel is assigned a photon. The advantages of this method are that it is fast (maximum linear run-time dependence on the photon count) and requires only two free parameters (*x*_seed_ and *x*_trh_), and thus features good robustness. The disadvantage is that higher photon counts are underestimated in comparison with lower counts.
